# Predicting Tree Species From 3D Laser Scanning Point Clouds Using Deep Learning

**DOI:** 10.3389/fpls.2021.635440

**Published:** 2021-02-10

**Authors:** Dominik Seidel, Peter Annighöfer, Anton Thielman, Quentin Edward Seifert, Jan-Henrik Thauer, Jonas Glatthorn, Martin Ehbrecht, Thomas Kneib, Christian Ammer

**Affiliations:** ^1^Faculty of Forest Sciences, Silviculture and Forest Ecology of the Temperate Zones, University of Göttingen, Göttingen, Germany; ^2^Forest and Agroforest Systems, Technical University of Munich, Freising, Germany; ^3^Campus Institute Data Science and Chairs of Statistics and Econometries, Göttingen, Germany

**Keywords:** machine-learning, artificial intelligence, tree species classification, laser scanning, convolutional neural networks

## Abstract

Automated species classification from 3D point clouds is still a challenge. It is, however, an important task for laser scanning-based forest inventory, ecosystem models, and to support forest management. Here, we tested the performance of an image classification approach based on convolutional neural networks (CNNs) with the aim to classify 3D point clouds of seven tree species based on 2D representation in a computationally efficient way. We were particularly interested in how the approach would perform with artificially increased training data size based on image augmentation techniques. Our approach yielded a high classification accuracy (86%) and the confusion matrix revealed that despite rather small sample sizes of the training data for some tree species, classification accuracy was high. We could partly relate this to the successful application of the image augmentation technique, improving our result by 6% in total and 13, 14, and 24% for ash, oak and pine, respectively. The introduced approach is hence not only applicable to small-sized datasets, it is also computationally effective since it relies on 2D instead of 3D data to be processed in the CNN. Our approach was faster and more accurate when compared to the point cloud-based “PointNet” approach.

## Introduction

Many functions and services of a forest are tied to forest structure and the structure of the individual trees that constitute it. Therefore, structural information is not only relevant for monitoring deforestation (Goetz and Dubayah, 2011), estimating carbon stocks (Asner, 2009) or predicting biodiversity (Bergen et al., 2009; Dees et al., 2012), but also to enable more accurate models of microclimatic conditions (Ehbrecht et al., 2017), the carbon cycle (Xiao et al., 2019), water cycle (Varhola and Coops, 2013), and other tasks. For an optimized and goal oriented forest management, detailed information on the stand structure is also essential. For example, to ensure habitat continuity (Delheimer et al., 2019; Franklin et al., 2019), to control fire risk (Hirsch et al., 2001) or to optimize timber yield (Kellomäki et al., 2019) and stand stability (Díaz-Yáñez et al., 2017).

Today, three-dimensional (3D) data of forests is available through terrestrial (e.g., Seidel et al., 2019), airborne (e.g., Abd Rahman et al., 2009; Vastaranta et al., 2013), and even spaceborne remote sensing platforms (e.g., Qi and Dubayah, 2016). High-resolution 3D data on individual trees is also available for larger areas (Koch et al., 2006; Liang et al., 2014) and provides the opportunity to aid research in forest ecology (Danson et al., 2018; Disney, 2019), tree architecture modeling (Bucksch and Lindenbergh, 2008; Dorji et al., 2019), and to support forest management in an unprecedented way (Hirata et al., 2009). However, two major challenges must be overcome if 3D data of forests is to be used operationally on a larger scale.

First, individual tree separation from the stand data must be fully automatized in order to make tree-based modeling possible. So far, most studies relied on manual selection procedures to cut individuals from stand-level 3D data in order to enable tree-based processing. This process may be very precise, since the human cognitive system does an excellent job in identifying 3D objects (Todd, 2004) and also proved to be quite reproducible (Metz et al., 2013), but it is also very tedious. Intensive research has tackled the challenge of automatic forest point cloud segmentation (Li et al., 2012; Lu et al., 2014; Ayrey et al., 2017) and recently commercial software has become available to do the task automatically, fully objectively and with remarkable success rates (e.g., software package “LiDAR360,” GreenValley International, Berkeley, CA, United States^1^).

The second challenge lies in the automatic classification of identified tree individuals with regard to their species. This is the focus of the study presented here.

Tree species information is often a crucial parameter in forest inventory, for ecosystem models, or for forest management (Terryn et al., 2020). There have been several successful attempts to determine tree species solely based on structural attributes from high-resolution ground-based LiDAR data. While some studies used selected measures describing tree architecture to predict the species (Åkerblom et al., 2017; Terryn et al., 2020), others used bark characteristics (Othmani et al., 2013) or combinations of several selected structural measures like tree height, leaf area index, branch angle (Lin and Herold, 2016). In the latter study, combinations of as many as ten structural features proved very successful when predicting the tree species from 3D data. Some studies attempted the species classification task using deep learning techniques. For example, Guan et al. (2015) applied deep learning methods in order to classify tree point clouds collected using mobile laser scanning data in the roads of Xiamen City, China. Their algorithm included preprocessing steps, for example, ground points from the road surface were removed from the 3D representations. Trees were then individually segmented and the algorithm extracted geometric structures, more precisely waveform representations, of the single trees, to classify each individual using a support vector machine classifier. This strategy was applied to ten different tree species including 50,000 samples for training. In order to test their algorithm, they used more than 2000 tree individuals covering the same ten species and attained a classification accuracy of 86.1% (Guan et al., 2015). Terryn et al. (2020) also used support vector machines to classify tree species with mean test accuracies of around 80%. The latter study reported difficulties due to increased intra-species variability caused by size differences of the sampled trees as well as due to convergent structural traits across species for individuals of the same canopy class and shade tolerance group (Terryn et al., 2020).

The recent surge in availability of 3D models has led to various advancements in the development of 3D classification models (Qi et al., 2016). Several different approaches to process 3D objects like chairs vs. tables exist, including direct use of unordered point clouds, or using artificial neural networks that work on volumetric object representations (Maturana and Scherer, 2015; Ben-Shabat et al., 2017; Qi et al., 2017a, b). While there are neural networks that are able to classify 3D point clouds with promising accuracies, compared to 2D image classification, the results are still unsatisfactory. The reasons for that are diverse. One major and recurrent problem is the unordered nature of point clouds, as differently ordered points still depict the same point cloud, resulting in *N!* possible representations of the same point cloud (*N* = number of points). Another common problem when trying to accurately predict species from point clouds is that point clouds depict objects that usually differ in size. In the particular case of 3D point clouds of trees from terrestrial laser scanning there is the additional problem of a rather small sample size in terms of tree number in most studies. In contrast, airborne laser scanning (ALS) campaigns may produce point clouds of hundreds or thousands of trees, and some pioneering studies reported successful classifications of tree species directly from the point cloud (Budei et al., 2018). Classifications of deciduous/coniferous trees were also successful using airborne data (Hamraz et al., 2019). First approaches from mobile laser scanning have been mentioned above, but only for urban trees (Guan et al., 2015). With regard to TLS, Zou et al. (2017) introduced an approach for species classification that reached up to 95.6% accuracy, using automatically extracted individual 3D tree point clouds that were transformed into 2D images before classification into four different species. Similarly, Mizoguchi et al. (2019) performed a transformation of 3D point clouds into images in order to facilitate classification tasks based on the bark surface of two species. They reached classification accuracies also often greater than 90%. Despite these promising results, in a recent study, it was argued that 3D to 2D transformations come with the cost of a considerable loss in 3D structural information (Xi et al., 2020).

This seems intuitively right, but 2D data is not only processed much faster than 3D data, most classification algorithms directly using raw point clouds also achieve considerably worse results than e.g., 2D image classification (Ben-Shabat et al., 2017; Qi et al., 2017a, b). Therefore, it is interesting to explore the 2D classification approach further.

Convolutional Neural Networks (CNN), first introduced by LeCun et al., 1995, are particularly suited for image classification, as each neuron in the network is only connected to a limited number of other neurons (sparse connectivity). Furthermore, CNNs share parameters efficiently (Goodfellow et al., 2016). In contrast to using raw point clouds, images are regularized input data, as they are easily standardized to have the same amount of pixels to be evaluated. The problems that come along with having to be invariant to *N*! permutations are thus mitigated.

Additionally, the analysis of regions that lie adjacent to one another is much simpler in an ordered 2D environment. The ordered nature of images is perfectly exploited by CNN’s, as “subpictures” of the input images are taken and connected (via kernels) to single elements of matrices in the following layer, connecting only adjacent pixels in this layer.

The vulnerability of the CNN approach to aspects such as size and position of objects in the images is thus reduced (LeCun et al., 1998), allowing easier analysis of positional and dimensional pattern when compared to the point clouds. The analysis of neighboring regions is intuitively much more difficult in 3D where the same region can be represented in *N!* different ways. In case of trees, such variations include the position of different branches or the curvature of the stem. When looking at single batches of images much more compressed information is provided when compared to looking at batches of 3D point clouds, as adjacent pixels are more correlated than pixels further apart (Raschka and Mirjalili, 2019).

Motivated by the above, we tested the performance of an image classification approach based on CNN with the aim to classify seven tree species from 2D representations (images) of 3D point clouds. We were particularly interested in how the approach would perform with and without artificially increased training data size, which we created through “augmented” (slightly altered) images. This issue may be particularly important for studies that only provide a small sample of 3D point clouds per species. For comparison of our findings with an existing point cloud-based approach, we also applied the PointNet-approach to our 3D data.

## Materials and Methods

### Laser Scanning and 3D Tree Point Clouds

3D tree point clouds used in this study (*n* = 690) originated from several laser scanning campaigns conducted in the last decade. All scans were captured in forest sites located in Germany and the United States (Oregon), including managed as well as unmanaged forests (National Parks). The sampled sites represent a large variety in soil conditions, climatic characteristics, and management regimes. All scans were made with a Faro Focus 3D 120 (Faro Technologies Inc., Lake Marry, FL, United States) or a Zoller and Fröhlich Imager 5006 (Zoller and Fröhlich GmbH, Wangen i.A., Germany). Details on the devices, scan settings, and environmental conditions at the various studies site are provided in the original studies cited in Table 1. However, some key information is given in the following for a better understanding of the data used.

**TABLE 1:** Overview on the datasets used in this study with the number of tree point clouds per species, reference to the original studies for more detail and the scan devices used.

Species	Number of trees	Original study	Scanner
Beech	163	Seidel et al., 2019	Z + F
		Metz et al., 2013	Z + F
		Seidel et al., 2011	Z + F
		+unpublished data^1^	Faro
Red oak	100	Burkardt et al., 2019	Faro
Ash	39	Seidel et al., 2011	Z + F
		Metz et al., 2013	Z + F
		Seidel et al., 2019	Z + F
Oak	22	unpublished data^2^	Faro
Douglas Fir	183	Seidel et al., 2016	Faro
		+unpublished data^1^	Faro
Spruce	158	Metz et al., 2013	Z + F
		+unpublished data^1^	Faro
Pine	25	Metz et al., 2013	Z + F
		+Unpublished data^1^	Faro
Total	690		

*^1^Data from research sites of the Research Training Group 2300 (https://www.uni-goettingen.de/en/574316.html). ^2^Data from the DE5 research site introduced in Pretzsch et al., 2020.*

The angular scan setting was the same for all scans (0.035° or 10285 points per 360°) and for both scanners. Minimal scan distance 0.6 m and 1 m for the Faro and Imager, respectively. Maximum scan distance was 120 m for the Focus 3D and 79 m for the Imager 5006. All trees were placed within the actual scan ranges of the scanners (never closer, never further away). However, the scanner-to-tree distance differed among the trees since all trees were separated from multi-scan approaches covering larger forest areas. There were always at least four (max.: 17, mean: 7) scans capturing a tree form varying directions and distances. Naturally, co-registered point clouds had a notable variation in point cloud densities due to the different amounts and distances of scans contributing to the point cloud of an individual. Since low-resolution images were created from the point clouds in the following (see next chapter), we dispensed further point cloud standardization based on point cloud density. In fact, point cloud density was much larger in all cases than what could be depicted in the images made from the point clouds. Therefore, the below described conversion of the point-clouds into low-resolution images resulted in a drastic standardization in both, resolution and density of the data before it was used further.

The beam footprint of the two devices was below 1 cm up to a distance of 31.82 m for the Imager and 38.75 m for the Focus, respectively. We therefore assume the effect of footprint size to be neglectable in the data.

Those datasets that were not published so far originated from two different studies conducted in Germany (see details provided in Table 1). For the present study, we ignored effects of different origins of the trees and assumed the scans comparable from the two scanner models. Despite almost identical scan settings it is, however likely, that the two scanner models and the variable scan design in the field (incl. different numbers of scans per tree) resulted in some specific characteristics of the point clouds, for example different amounts of stray points and different point densities, as already mentioned. To further standardize the data, all scans were post-processed and filtered for erroneous points as described in the original studies, which included filters for isolated points, points with unclear reflection pattern (too dark, too bright) and points resulting from split laser beams. The softwares provided by the manufacturers of the two scan devices automatically removed all these point and we used the standard settings provided in the Faro Scene software (Faro Technologies Inc., Lake Marry, FL, United States) and the Z + F lasercontrol software (Zoller und Fröhlich GmbH, Wangen, Germany).

**FIGURE 1:**
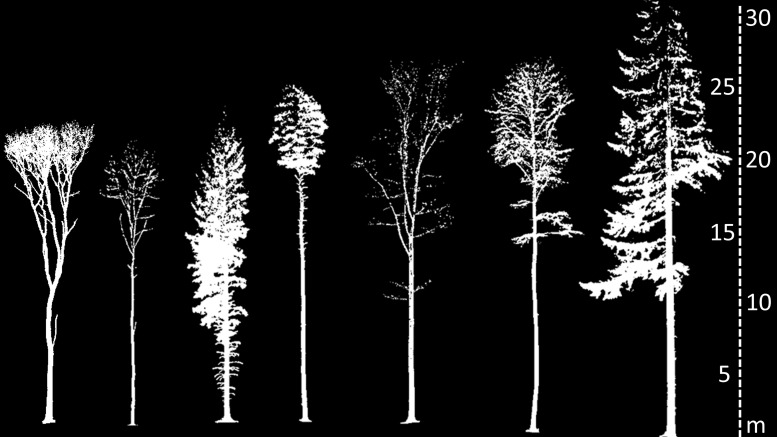
2D representations of 3D point clouds of an exemplary tree of each studied species. From left to right: Sessile oak (*Quercus petraea* L.), European ash (*Fraxinus excelsior* L.), Norway spruce (*Picea abies* L.), Scots pine (*Pinus sylvestris* L.), red oak (*Quercus rubra* L.), European beech (*Fagus sylvatica* L.), and Douglas-Fir [*Pseudotzuga menziesii* (Mirbel) Franco]. Trees are in scale (see scale bar on the right).

In a next step, each tree individual was manually separated from the forest point cloud using 3D visualization software as described in Metz et al. (2013). To do so, Scans obtained from the Faro Scanner were processed using CloudCompare (www.danielgm.net) and scans obtained with the Imager 5006 were processed using Leica Cyclone software (Leica Geosystems AG, Heerbrugg, Switzerland). All individual tree point clouds were exported as 3D point clouds in xyz-file format for further processing. An overview of an exemplary tree for each species considered in our study is provided in Figure 1.

### Data Processing

Typical convolutional network architectures require highly regular input data formats like those of 2D grids or 3D voxels to perform weight sharing and other kernel optimizations. To enable the use of image recognition and classification approaches, we transformed the 3D point clouds into image representations. 2D representations in the form of images are ordered by nature, since a different pixel arrangement does not lead to the same representation of the object. Also, the different sizes of the matrices do not play a role in images because the size of the images is defined by the chosen number of pixels and can be set identically for all images. To transform point clouds into images we plotted a randomly selected sample of 6,000 points from each tree’s point cloud to create a scatterplot. In this approach, caution is advised since small trees may be represented in more detail than large trees if size differences are profound. Therefore, we compared only adult forest trees. The scatterplot was then saved as an image with 150 × 100 pixels which are rather large images for image classification problems. Common image sizes from popular benchmark datasets were 28 × 28 or 32 × 32 pixels (Deng, 2012). We tested differently sized images, but found that having less pixels in the images led to poor representations of the trees.

**FIGURE 2:**
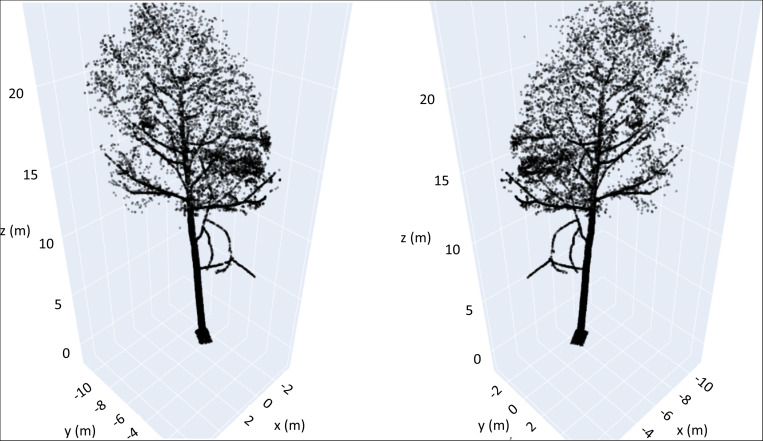
3D scatterplots of an exemplary beech tree from two different perspectives (180° of rotational difference in the xy-plane).

For each tree’s point cloud we repeated the process for different viewing angles (rotation in the xy-plane, see Figure 2 for an example) to minimize the information loss of changing from a 3D- to a 2D representation.

**FIGURE 3:**
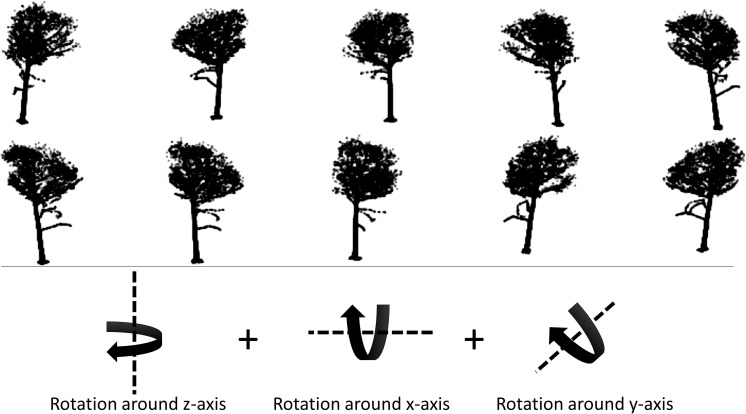
Illustration of augmented point cloud views on the beech tree shown in Figure 2 from ten different viewing angles with rotations of the point cloud around all three axis combined. Additionally, we applied small vertical and horizontal shifts, added Gaussian noise, image sharpening and a change of contrast but those are hardly notable in the images.

The parameters to be determined were therefore the number of “screenshots” per tree and hence the degree of rotation after which a new image should be produced and the pixel size of the saved images. In order to avoid oversimplifying our dataset and thereby inducing overfitting into our model, we chose a fairly conservative approach of generating ten 8-bit grayscale images per point cloud, as illustrated in Figure 3. Since the scatterplots had constant marker-sizes for points throughout the 3D space (with filled circular markers of fixed size independent from the viewpoint on the scatterplots), the 256 different gray values of the 8-bit images were used to reflect locational differences (lighter gray values for points further away).

Unfortunately, the dataset was not balanced with regard to the number of samples per tree species. As it was established that imbalanced datasets can have a significant negative impact on training classifiers (Japkowicz and Stephen, 2002), we needed to adjust the dataset when trying to classify all subspecies. We generated additional tree images for those species that were underrepresented, such as European ash, Scots pine, Norway spruce and Douglas-fir. To do so, we made use of classical data augmentation techniques. The datasets were thereby extended with newly generated, plausible examples. The transformations we applied were a weak rotation, a small vertical and horizontal shift, added Gaussian noise, image sharpening and a change of contrast. Those techniques were applied to all species, but we enhanced the number of underrepresented trees more severely. We did not try to create a completely balanced dataset as we would either have to delete additional trees, thus reducing the size of the dataset further, or create a large amount of images from the more scantily available species, thereby reducing the datasets variance and risking oversimplification of the dataset. We conducted a strict split of training and test data prior to data augmentation in order to avoid any overlap between training data and those images used for testing the approach. Each tree’s images were either completely in the training dataset or completely in the test data based on a random assignment of individuals.

### Image Classification Using a Convolutional Neural Network

We chose a fairly simple and easy to implement network architecture, closely resembling the LeNet 5 architecture introduced in LeCun et al. (1998). We implemented the network using Keras (Chollet, 2015).

The network consists of four convolutional-, four maxpooling- and two dropout layers. Across the entire model, we used a fixed kernel size of 3 × 3. The kernels were all applied to the images in small 2D windows. The chosen filter size seems rather small for the large size of the input images of 150 × 100 pixels. However, the 5 × 5 filters used in LeNet 5 did not perform as well as the 3 × 3 filters. The output of a convolutional layer, consisting of multiple kernels, are multiple feature maps. These maps are two dimensional arrays. Throughout the complete network we used the Rectified Linear Unit (ReLU) activation function [*f*(x) = max(x, 0)].

The 150 × 100 input images were fed into the first convolutional layer using eight filters with a stride size of one. The first convolutional layer is followed by the first maxpooling layer with receptive fields of size 2 × 2 and a stride size of one pixel and the purpose of shrinking the size of the respective feature map and reducing the complexity of the model (O’Shea and Nash, 2015). Furthermore, positional invariance over local regions is enabled.

Although images do not induce the same problems as unordered point clouds, it is desirable to be invariant to certain positional invariances in the images. In the maxpooling layers, the feature maps are processed one small field at a time. The elements of one field are pooled using the maximum function, as Scherer et al. (2010) found that maxpooling can lead to faster convergence. The subsequently following second convolutional layer consists of sixteen 3 × 3 filters and is again followed by a maxpooling layer. The third convolutional layer consists of 32 3 × 3 filters and is followed by the first dropout layer. The 0.3 dropout layer had the purpose of reducing overfitting problems and in line with Hinton et al. (2012) drastically improved the model’s performance. Generally, dropout has the effect of forcing units within a layer to probabilistically take on more or less responsibility for given inputs. Feature detectors were deleted during training (Baldi and Sadowski, 2013) with the predetermined probability of 30%. To compensate the loss of these feature detectors, the remaining feature detectors needed to be adjusted to obtain continuously accurate prediction results, thus successfully generalizing the given input images. The 0.3 dropout layer was followed by the last convolutional layer of 64 3 × 3 filters. We subsequently flattened the 64 feature maps and led them into a fully connected layer consisting of 128 neurons. The fully connected layer was followed by the second dropout layer, this time deleting feature detectors during training with a probability of 50 percent. The last fully connected layer in our model, the classification layer, has seven output units, as we classified seven different tree species.

For the final output layer, we used a different activation function, namely the softmax activation function which transforms the input vector to a probability vector. Thus, we used the commonly used cross-entropy loss function, strongly penalizing bad predictions (Géron, 2017) and optimized the models loss using the Adam optimizer.

The models’ output were thus specific probabilities for each tree, expressing the certainty for a label prediction (Buduma and Locascio, 2017). The accuracy of the model was evaluated with an independent test dataset. From the full set of trees, some trees and the corresponding images were randomly selected for testing (Table 2). The species of each tree in the test dataset was predicted with the model and compared to the actually observed tree species. To obtain a unique classification, the species with the highest probability was always selected.

**TABLE 2:** Number of images created through rotational views on the point clouds as well as added images through the augmentation.

Species	Number of trees	Number of images	Number of images after augmentation	Number of images for training after augmentation	Number of images for testing after augmentation
Beech	163	1,630	1,630 (unchanged)	1,280	350
Red oak	100	1,000	1,000 (unchanged)	810	190
Ash	39	390	790	720	70
Oak	22	220	620	580	40
Douglas Fir	183	1,830	1,830 (unchanged)	1,460	370
Spruce	158	1,580	1,580 (unchanged)	1,270	310
Pine	25	250	650	600	50

*Note that images of an individual tree are always all in either training or testing data (train-test-split), never mixed.*

For comparison with an existing approach, we also tested the performance of the PointNet approach on our point clouds. While the original PointNet approach is based on 1024 points per input point cloud, we decided to use a higher number of points for the difficult task of classifying tree species. We were able to work with randomly picked number of 2048 points from each trees point cloud based on our computational resources. As the patterns and shapes that make the point cloud trees recognizable are more likely in the treetop, we decided to cut of the lowest 30% of points. Although this appears to be a lot, only about ten percent of the absolute height of the trees was actually cut, as the point density is naturally higher in the lower parts of the trees. This yielded significantly more recognizable representations of the trees, at least for the human eye. To increase the sample size, we repeated the random pick of points ten times per tree. Finally, a strict train-test-split was conducted again.

## Results

We successfully transformed the 3D point clouds into images by creating images from different perspective views on the point clouds. After image creation from ten perspectives per tree and additional image augmentation for underrepresented tree species, our data consisted of 4040 (50%) deciduous and 4060 (50%) coniferous tree images, with some images used for training and the remainder used for testing (see also Table 2). For comparison, Table 2 also shows the number of images used for testing the performance of the CNN approach without image augmentation.

**FIGURE 4:**
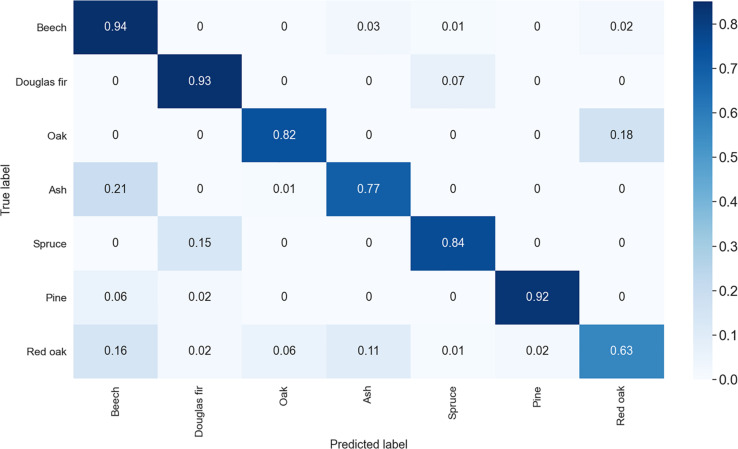
Confusion matrix of the species classification with image augmentation applied. Shown are the classification cases in decimals (times 100 = percent). The matrix is to be red from left to right (row wise) only.

Tree species classification based on our approach had an overall accuracy of 86.01% with augmentation applied. The confusion matrix (Figure 4) shows that the model very accurately classified Douglas-Fir trees (93% correct), Scots pine trees (92% correct), European beech trees (94% correct), and to some extent also Norway spruce trees (84%) and oaks (82%). The model was less accurate in the prediction of red oaks (63%) and ashes (77%).

**FIGURE 5:**
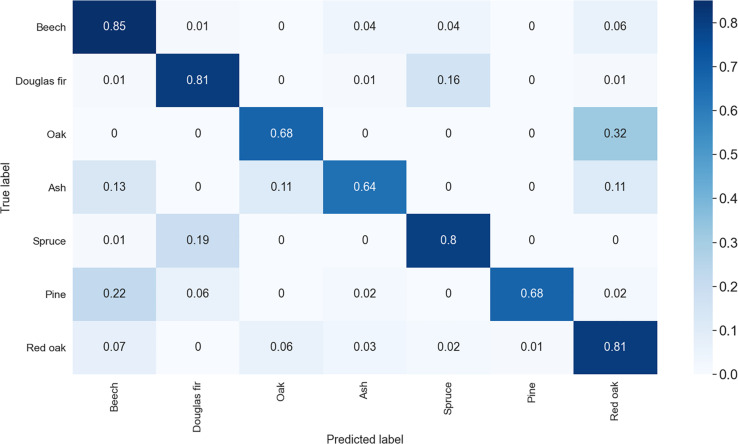
Confusion matrix of the species classification without image augmentation. Shown are the classification cases in decimals (times 100 = percent). The matrix is to be red from **left** to **right** (row wise) only.

With no augmentation applied, the overall accuracy dropped to 80.2% (Figure 5) with accuracies particularly dropping for those species with small initial sample sizes. Oak dropped to 68% accuracy, ash to 64% and pine to 68%. Surprisingly, for red oak, an increase from 63 to 81% accuracy was observed in the unaugmented dataset.

When it comes to confusion in the classification, it was found that for European beech confusion mainly occurred with European ash, while it was the other way around for ash (mostly confused with beech). Both species were also confused with oak and for European beech some confusion occurred also with Norway spruce. For Douglas-Fir and Norway spruce, we found that confusion mainly occurred both ways between these two species. Pine was in 6% of the cases misclassified as beech and in rarer cases as Douglas-fir (2%). Finally, red oak misclassification occurred with all other tree species, mostly though with other deciduous species (beech: 16%; oak: 6%; ash: 11%) and only in 5% of all cases with coniferous species (Douglas-Fir: 2%; spruce: 1%; pine: 2%).

Without augmentation, we observed greater confusion of species with small sample size (pine, ash and oak) with red oak and among each other.

**FIGURE 6:**
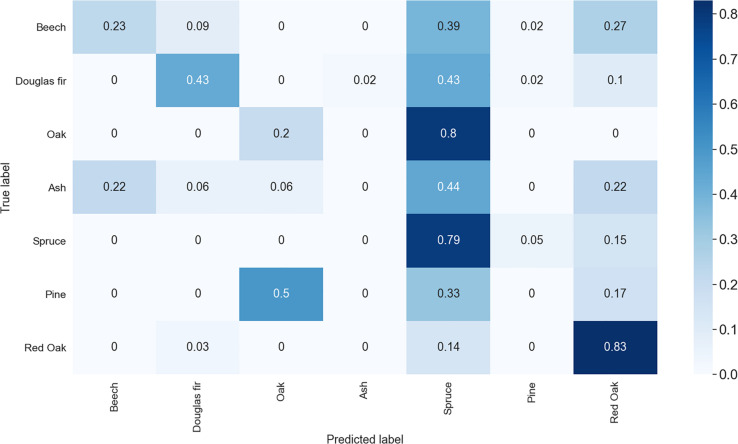
Confusion matrix of the species classification with PointNet based on the point clouds. Shown are the classification cases in decimals (times 100 = percent). The matrix is to be red from **left** to **right** (row wise) only.

Based on the PointNet approach, we did not achieve competitive accuracies. Classification accuracy was 23% for beech, 43% for Douglas-Fir, 20% for oak, 0% for ash and pine, 79% for spruce and 83% for red oak. The confusion matrix for the results obtained with PointNet is shown in Figure 6.

## Discussion

When dealing with LiDAR-based tree point clouds, classification of tree species is difficult since characteristics such as the tree bark or leaf structure, which are often used for classifications based on the human eye, are not necessarily available from a laser scan. While the bark structure may be a useful feature for species identification in very close-range scans (cf. Othmani et al., 2013; Mizoguchi et al., 2019) it may not appear in required detail at greater scanning distances. We are also not aware of studies that utilized solely leaf characteristics for tree species classification from laser scan data, even though leaf area index, a trait of all leaves together rather than individuals, was used in a pioneering study (Lin and Herold, 2016). In fact, leaf information from point clouds was denoted as “trivial” for species classification tasks (Xi et al., 2020), since classification should not depend on seasonality in the data (Hamraz et al., 2019). Furthermore, the spatial resolution of many laser scanners is not suitable to address detailed morphological differences among leaves. Hence, leaves, just like the bark, seem to be rather difficult to use for species classification from data obtained *via* terrestrial, mobile or airborne laser scanning in an operational way.

For these reasons, we decided to follow some pioneering studies by making use of the total tree architecture rather than specific structural elements. Puttonen et al. (2011) reported an accuracy of 65.4% when using LiDAR data from mobile laser scanning for tree species classification. Almost one decade later, Xi et al. (2020) conducted a benchmark test on the classification performance of several widely applied deep learning and machine learning algorithms that can be used for tree species classification and reported accuracies between 78 and 96% for nine tree species. Since the direct use of point clouds is computationally demanding, we followed a different approach based on image representations of the point clouds.

The overall accuracy of our approach was promising (86%) and we argue this is because the transformation of 3D to images enabled us to make use of the strong, already existing image classification techniques based on CNNs. In an attempt to compare our results from the CNN approach to the performance of the point-cloud based PointNet approach, we failed to achieve competitive results from the latter, all species considered. The small sample sizes for oak, pine and ash (<40 trees) may explain the bad performance for these species. For beech and Douglas-Fir the accuracies were low despite the larger sample sizes, particularly due to misclassification as spruce (see Figure 6). Overall, all tree species showed great confusion with spruce in the PointNet approach. Only for spruce itself and for red oak the PointNet approach yielded results in the range of the CNN-based approach. We have no explanation for the “attraction” of spruce or the high accuracy in the classification of red oak from the 3D data. However, the point cloud-based approach required a much greater computational effort and showed an overall weak performance on six out of seven tested species.

In contrast, we see great potential in the application of the CNN approach, particularly as it allows for additional image augmentation, which can strongly increase the sample size of the training data. This is crucial for small samples, like those for pine, ash and oak in our study. Such small sample sizes are not uncommon for terrestrial laser scanning campaigns in general. The observed overall difference in classification with and without image augmentation was 6% and largely attributed to a profound loss in classification accuracy of tree species with small sample sizes. While this clearly indicates the benefit of the augmentation approach, we also observed a surprising increase in accuracy for red oak (18%), a species that was not directly affected by the augmentation (no images augmented at all). When comparing the confusion matrices (Figures 4, 5) we can see that augmentation resulted in less confusion of red oak with ash and also with beech. A reduced confusion of red oak with ash is likely directly associated with the very low number of ashes in case of no augmentation and hence a reduced intra-species variability in the data of ash. The observed reduction of confusion of red oak with beech cannot be directly explained with augmentation, since images of neither of the species were augmented. While it is difficult to associate differences in prediction accuracies of two different CNNs directly to a specific cause, we hypothesize that in our case the differences are most likely attributed to a different training data set picked during the random train-test-split. If datasets are small (red oak = 100 trees) the effect of randomly picked training data can be fairly large, in our case a reduction in confusion of red oak with beech of 9%.

In general, we found that if the input data (of a given class; here: species) for the training of the CNN contained trees from a broad spectrum of growing conditions (here: red oak and beech) and the training dataset was rather small at the same time, classification success rates were lower compared to other species. Varying site conditions, management approaches, stand ages, and stand densities in each location resulted in different phenotypes of trees of the same species, but those trees were of course assigned to one class (here: red oak). This affects the accuracy of each species’ classification differently, since each species’ data originated from a varying amount of study sites and varying degrees of heterogeneity among the study sites. However, despite the small training data sample size of pine, this species was classified with high accuracy. We argue, this is because all Scots pine trees originated from a single study site with homogenous growing conditions for all trees.

Another reason for a confusion during the classification may be that species have a rather strong morphological resemblance. For example, Douglas-Fir was confused with Norway spruce and vice versa, which seems reasonable given the similarities in overall tree shape. Again, the small sample of pine trees was likely morphologically homogenous enough to allow for a very high classification accuracy despite small data size. This indicates a low variability in our dataset of pine trees, which is in contrast to that of beech (many different kinds of stands), Douglas-fir (Germany and United States) or red oak (ten different sites in Germany; cf. Burkardt et al., 2019). While the large sample sizes of these species may have compensated the negative effect of a large variety of tree shapes within a class, red oak classification accuracy was still rather low (Figure 4). Here, confusion occurred with all other species, but mostly with other deciduous species. We argue that this can be explained with the strong morphological differences among the observed 100 red oak trees, originating from ten different study sites (ten individuals per cite) within different management histories and distributed across Germany.

Considering the above, we see great potential for the approach presented here. Image augmentation can be used to enhance the dataset when sample sizes are small and the image-based approach can be used whenever computational efforts must be kept at a minimum.

We recommend using a standardized scan setting during data acquisition, with both scan resolution and number of scans per tree as constant as possible. Furthermore, it is advised to apply identical post-processing steps (tree segmentation, filtering) to all trees used in a study. We finally recommend to avoid applying our approach to trees of vastly different sizes, such as juvenile trees and mature trees at the same time. This may result in different levels of detail represented in the images and consequently affect the classification accuracy.

## Conclusion

We conclude that the presented data transformation and image classification methods seems to be a valid approach for classifying 3D point clouds of trees with regard to species based on CNNs and image augmentation. We found that a classification accuracy of 86% for the seven tested tree species was possible despite small initial sample sizes and remarkable variation in the morphologies within tree species classes. Only in cases were both issues were present, namely a large within-species morphological variability and a small sample size, we observed lower classification accuracies. The PointNet approach used for comparison suffered from the small sample size and did not yield a competitive classification accuracy on our data, despite much greater computational effort.

Our approach of removing one spatial dimension from the initial data may come at the cost of a loss in characteristics that may be helpful for the classification task. However, using 2D representations created from different perspectives on the original 3D objects may have reduced such a loss of information. At the same time, the capabilities of existing deep learning algorithms with regard to 2D image classifications are remarkable and became only available through the reduction in dimension. Improvements of the classification accuracies for selected species could likely be achieved when a larger dataset can be used for training, since low accuracies were, with the exception of pine, associated with small sample sizes. This leads to the conclusion that small sample sizes are not necessarily a problem if the properties of the object class (here the structure of pine trees) are remarkable enough. Together with available automated tree segmentation approaches, we see great potential for operational use of the presented method in future forest inventories.

## Data Availability Statement

The datasets presented in this study can be found in online repositories. The names of the repository/repositories and accession number(s) can be found below: https://data.goettingen-research-online.de/dataverse/gro. The datasets for this study can be found here: https://doi.org/10.25625/FOHUJM.

## Author Contributions

DS, PA, JG, TK, and CA: conceptualization. DS and ME: data acquisition. DS, AT, QS, and J-HT: data processing. DS: manuscript writing. DS and CA: funding. All authors contributed to the article and approved the submitted version.

## Conflict of Interest

The authors declare that the research was conducted in the absence of any commercial or financial relationships that could be construed as a potential conflict of interest.

## Footnotes

1 https://greenvalleyintl.com
